# New inflammatory indicators for cell-based liquid biopsy: association of the circulating CD44+/CD24− non-hematopoietic rare cell phenotype with breast cancer residual disease

**DOI:** 10.1007/s00432-022-04330-5

**Published:** 2022-09-13

**Authors:** Stefan Schreier, Prapaphan Budchart, Suparerk Borwornpinyo, Wichit Arpornwirat, Panuwat Lertsithichai, Prakasit Chirappapha, Wannapong Triampo

**Affiliations:** 1 School of Bioinnovation and Bio-Based Product Intelligence, Faculty of Science, Mahidol University, Rama VI Rd, Bangkok, 10400 Thailand; 2 Thailand Center of Excellence in Physics, Ministry of Higher Education, Science, Research and Innovation, 328 Si Ayutthaya Road, Bangkok, 10400 Thailand; 3 Department of Physics, Faculty of Science, Mahidol University, Bangkok, 10400 Thailand; 4 Premise Biosystems Co. Ltd, Bangkok, 10540 Thailand; 5 Excellent Center for Drug Discovery, Faculty of Science, Mahidol University, Rama VI Rd, Bangkok, 10400 Thailand; 6 Department of Oncology, Bangkok Hospital, 2 Soi Soonvijai 7, New Petchburi Rd, Huaykwang, Bangkok, 10310 Thailand; 7 Department of Surgery, Faculty of Medicine Ramathibodi Hospital, Mahidol University, Bangkok, 10400 Thailand

**Keywords:** Systemic inflammation, CD44, Circulating rare cells, Liquid biopsy, Minimal residual disease, Breast cancer

## Abstract

**Background:**

Breast cancer residual disease assessment in early-stage patients has been challenging and lacks routine identification of adjuvant therapy benefit and objective measure of therapy success. Liquid biopsy assays targeting tumor-derived entities are investigated for minimal residual disease detection, yet perform low in clinical sensitivity. We propose the detection of CD44−related systemic inflammation for the assessment of residual cancer.

**Methods:**

Circulating CD44+/CD45− rare cells from healthy, noncancer- and cancer-afflicted donors were enriched by CD45 depletion and analyzed by immuno-fluorescence microscopy. CD44+ rare cell subtyping was based on cytological feature analysis and referred to as morphological index. AUC analysis was employed for identification of the most cancer-specific CD44+ subtype.

**Results:**

The EpCam−/CD44+/CD24−/CD71−/CD45−/DNA+ phenotype alludes to a distinct cell type and was found frequently at concentrations below 5 cells per 5 mL in healthy donors. Marker elevation by at least 5 × on average was observed in all afflicted cohorts. The positive predicted value for the prediction of malignancy-associated systemic inflammation of a CD44+ rare cell subtype with a higher morphological index was 87%. An outlook for the frequency of sustained inflammation in residual cancer may be given to measure 78%.

**Conclusion:**

The CD44+ rare cell and subtype denotes improvement in detection of residual cancer disease and may provide an objective and alternative measure of disease burden in early-stage breast cancer.

## Introduction

Despite progression in the efficacy of breast cancer therapy over the past decades, a considerable chance of relapse persists in high-risk patients with apparently node-negative localized disease (Coates et al. 2015; Gamucci et al. 2013; Pan et al. 2017; Bradley et al. 2021). The decade-old explanation of in particular distant relapse in localized early-stage cancer maintains the notion of low-grade systemic disease that persists without illness after complete resection of the tumor or surrounding lymph nodes then, referred to as minimal residual disease (MRD) (Riethmüller and Johnson 1992). MRD has been traditionally related to residual in situ tumor lesions and to residual clonogenic tumor cell populations forming locally occult micro-metastasis at a distant site foremost in the bone marrow or lymph nodes (Cote et al. 1999; Braun et al. 1999; Pantel and Alix-Panabières 2019). More recently, MRD was associated with the presence of tumor-derived material in the blood circulation in form of circulating tumor cells (CTC) or circulating tumor DNA (ct-DNA) arriving at the conclusion of occult active disease and a persisting systemic cancer disease character (Pantel and Alix-Panabières 2019).

Apparently, assessment of residual cancer disease particularly in declared complete responders would resolve a diagnostic void and potentially support individualized evidence-based decision making for adjuvant therapy mode and/or protocol, provide a measure of individual objective response, and of therapy success and advice on diagnostic follow-up schemes. The most common platforms to support MRD assessment are liquid biopsy assays targeting CTC or and/or ct-DNA. However, circulating tumor-derived material, particularly CTC are expected to decline or vanish upon residual or totally resected tumors given the diminished source, a relative low sensitivity towards residual micro-lesions and the alleged half-life of at best a few hours in the circulation (Meng et al. 2004; Williams et al. 2020). Commonly and as can be expected, the clinical sensitivity towards early-stage breast cancer MRD did not exceed 30% when tested by various fully validated liquid biopsy systems (Pantel and Alix-Panabières 2019). The low sensitivity may not reflect the biological reality and would explain the relative low at best, inconsistent predicted values for recurrence particularly, in CTC positive cases (Pantel and Alix-Panabières 2019). Potentially, more sensitive and reliable markers may be needed for the MRD assessment and could be explored in the less investigated field of cancer systemic inflammation. In general, systemic inflammation plays a vital role in tumor evolution (Roxburgh and McMillan 2014) and consequently, denotes an important and perhaps more dominant aspect of MRD (Murray et al. 2021; Cole 2009). Cancer-associated systemic inflammation has been largely illuminated as immune-inflammation using traditional immune-associated markers that may include white cell and platelet counts, acute phase proteins, and their combinations. Nevertheless, traditional markers are not in routine use for breast cancer MRD assessment for reasons of a low positive predicted values (PPV) particularly in afflicted individuals with symptom-less inflammation. Alternatively, a spectrum of non-hematopoietic cell types are known to play role in chronic inflammatory processes and include parenchymal (Puré and Cuff 2001; Burger and Touyz 2012), endothelial (Lin et al. 2021; Hida and Klagsbrun 2005; Bhakdi, et al. 2019), stromal (Galland and Stamenkovic 2020) or mesothelial cells (Yung and Chan 2009). Awareness is growing about some of those inflammation-associated non-hematopoietic cell types to circulate in the blood stream as a consequence of recruitment presumably from the bone marrow or as a consequence of detachment from a tissue lesion (Lin et al. 2021; George et al. 2004; Schreier and Triampo 2020, 2021a). More recently, our group intended to introduce the circulating erythroblast and their aberrations to represent bone marrow damage aimed at the assessment of systemic cancer (Schreier et al. 2021b). Such inflammation-associated circulating rare cell types represent valuable diagnostically exploitable abnormality that may persist as residual cancer disease and could be denoted as the circulating rare cell cancer inflammasome.

The before mentioned set of CRC that are known to be associable with systemic cancer is limited and represents localized or specialized representatives of inflammation. For example, vascular inflammation is represented by circulating endothelial cells or bone marrow damage by circulating erythroblasts. Major pieces of the puzzle are still missing and can perhaps be found in markers directly involved in systemic inflammation. Such a suitable marker might be related to the CD44 antigen (Johnson and Ruffell 2009; Nikitovic et al. 2015). The CD44 antigen has been traditionally used in liquid biopsy as lead marker to identify tumor-derived epithelial circulating tumor stem cells based on its association with multi-lineage differentiation and the potential of self-renewal (Theodoropoulos et al. 2010). However, the marker has its place also in systemic inflammation as cell adhesion molecule and its principal ligand hyaluronan (HA), being expressed on leukocytes, epithelial (Goodison et al. August 1999), endothelial (Trochon et al. 1996), mesenchymal (Herrera et al. 2007) and mesothelial cells (Gardner et al. 1995). Inflammation that involves CD44 comprises in parts CD44–HA interactions that alternate cell functionalities at the site of lesion or injury and has been implicated in autoimmune diseases and cancers (Nikitovic et al. 2015).

In this respect, we intend to introduce a so far not investigated and distinct inflammation-associated circulating rare cell phenotype herein denoted as the non-hematopoietic CD44−positive circulating rare cell (CD44+ CRC) which, can be identified using the CD326−/CD71−/CD44+/CD24−/CD45−/Hoechst+ phenotype. We intended to showcase a simple approach of marker development for the application in cancer liquid biopsy. Similar to the behavior of before mentioned inflammation-associated rare cells, the CD44+ CRC also showed what we have referred to as multi-pathology association (Schreier and Triampo 2021a) which, in consequence translates into a low specificity towards cancer. An in-house subtyping approach based on cyto-morphological classification was employed to increase specificity towards malignancy and was found useful in the detection of residual cancer disease. Marker validation followed common strategy of accuracy analysis based on the area under (a ROC) curve analysis.

## Materials and methods

### Blood donations

The presence of CD44+ cells was tested in random donor selection by liquid biopsy on 10 healthy individuals denoted as healthy cohort (average age 40.9), 4 non-malignant individuals denoted as noncancer afflicted cohort (average 45.8 age) and 9 early-stage breast cancer patients denoted as donor cancer-afflicted cohort (average age 56.4). The latter cohort was formerly diagnosed with invasive ductal carcinoma, staged T1-2, N0-1 and M0. Blood donations by the malignancy cohort were classified into pre-surgery, post-surgery (MRD baseline), mid-term adjuvant therapy and completion. Donations of the noncancer cohort comprised a blood sample from a female individual 1 week after having received her 1st Pfizer anti-Covid 19 vaccination, a male individual diagnosed with low-grade non-alcoholic fatty liver, a blood sample from an individual with “unwell-feeling” including early night-time fatigue, short breathness, nocturnal nausea and liquid biopsy diagnosis of endothelial dysfunction, and a blood sample from a chronic diabetes type II patient. The negative control cohort was defined as being unaware of chronic underlying diseases, having been disease-free within the past 2 months, and not requiring any medication.

Patient blood was drawn on the day before onset of any therapeutic activity such as the administration of standard adjuvant chemotherapy or surgery.

### MRD detection platform

The cell-based liquid biopsy platform comprised whole blood pre-enrichment removing bulk red blood cells, nucleated cell enrichment removing bulk white blood cells and automated fluorescence microscopy for analysis. In detail, peripheral blood was taken by venous puncture collecting 10 mL in a green-top BD Vacutainer blood collection tube containing sodium heparin. The blood sample was kept at room temperature (RT) in the dark and processed at most 3 h after phlebotomy. Informed consent was sought from the patient at the time before the blood draw. Red blood cell lysis was required for the removal of erythrocytes. Standard chemical lysis buffer (154 mM NH_4_Cl, 10 mM NaHCO_3_, 2 mM EDTA) treatment was applied to remove red blood cells (RBC) from 10 mL whole blood. The cell suspension was incubated twice at RT for max. 3 min following centrifugation at 300×*g* for another 5 min each. The final cell pellet was resuspended in 1 mL PBS, supplemented with 0.5% bovine serum albumin and allowed resting for 15 min at room temperature. The cell numbers of nucleated cells subsequent to RBC lysis were determined by hemocytometer (Neubauer) and subjected to enrichment. In brief, peripheral blood rare cell isolation was carried out by automated CD45 positive cell depletion assay (Walderbach II) following the manufacturers description (SanoLibio GmbH, Germany). Subsequent to enrichment, the sample was split into halves containing 30 µL each and immediately stained for subsequent analysis by fluorescence microscopy adding anti-CD45PE (ebioscience), anti-EpCamFITC (ebioscience) and anti-CD44Superbright645 (invitrogen) in one panel, and combining anti-CD44Superbright645 (invitrogen) anti-CD24 PE(ebioscience) and anti-CD71FITC (ebioscience), CD45PercPCy5.5 (ebioscience) in the second panel, for each using 0.5 µL undiluted dye solution and incubating at RT in the dark for 25 min. A washing step followed by diluting the cell suspension with 1 mL PBS, supplemented with 0.5% bovine serum albumin prior to pelleting by centrifugation at 300×*g* for 3 min and resuspending in 120 µL using PBS. Nucleus staining followed using 0.5 µL Hoechst 33342 DNA staining (ThermoFisher). Both sample cell suspensions were then loaded and spun down at 80×*g* for 2 min in one well of a specialized 386-well plate (PerkinElmer) suitable for high-resolution image recording at 40× magnification using the Operetta system (PerkinElmer). Images were recorded in a bright-field channel, and channels for UV, green, yellow, orange, and red fluorescence emission. Columbus analysis software served as a screening and image analysis tool. Rare cell marker positive cells were identified by a cell-like round formation identifiable by morphology, positive Hoechst staining, and positive green (CD326 or CD71) and/or orange (CD44) fluorescence emission in the absence of the typical ring formation or membrane staining as a consequence of positive CD45PE or CD45PerCy5.5 staining.

### Imaging analysis

Each cell of interest was attributed with a value of the morphological index that was established according to the following procedure:

(i) Assessment of positivity: In order to be classified as CD44+ phenotype of interest, fluorescence emission needed to range at background noise levels in the green (EpCam or CD71), yellow (CD45 or CD24) and red channels (CD45), respectively. CD44 fluorescence signal needed to correspond to cell circumference according to the bright-field image and 3SD levels above mean background noise then alleging the EpCam−/CD71−/CD24−/CD45−/CD44+/Hoechst+ phenotype of interest.
(ii) Measurement of maximal CD44+ and Hoechst fluorescence emission intensity: highest fluorescence pixel intensity of the CD44+ signal was measured at the location of bright-field cellular circumference, strictly excluding the nucleus area. Respective background fluorescence noise was measured in 3 locations in vicinity of the cell event and averaged for both CD44 and Hoechst channels. The ratio between maximum intensity and background intensity values denoted the CD44 and Hoechst intensity ratio, respectively, and was used for further calculations of the morphological index.
(iii) Cell shape classification: the cell shape as it appeared under bright-field and CD44 fluorescence was classified into 3 morphological classes, one being round, a second being oval or slighted budded, and a third being irregular attributing each class with a 1 digit nominator, scoring 1, 2 or 3 and thus, and transforming the morphological information into the numerical space.
(iv) Calculus of the morphological index: a simple formula was used to define the morphological index as follows:
  $${\text{Morphological Index}} = N_{{{\text{Shape}}}}^{2} \, \times \,R_{{{\text{IntensityCD44}}}} \, \times \,R_{{{\text{IntensityHoechst}}}}$$

where *N*_shape_ denotes the shape class 1, 2, or 3, where *R*_IntensityCD44_ denotes the ratio between the maximum pixel value identified in the CD44 channel of the CD44−positive event and the averaged background value and where *R*_IntensityHoechst_ denotes the ratio between the maximum pixel value identified in the Hoechst channel of the CD44−positive event and the averaged background value, respectively.

### Statistical analysis

Each of the CD44−positive events was attributed with the morphological index as to support cyto-morphological subtyping of the CD44+ CRC. The purpose was to investigate the significance of the association between a particular CD44+ CRC subtype and chemotherapy-naive malignancy. The attempt to establish significance in the association followed common procedure of area under the ROC curve analysis and was performed via NCSS2022 Statistical Software. Sensitivity and 1 minus specificity data over a range of morphological index data were used to construct the ROC curves, and AUC was calculated with 95% CIs for sample classification variables. Higher AUC values represent greater accuracy. An AUC of 1.0 represents perfect sensitivity and specificity of the CD44+ CRC with a certain morphological index; an AUC of 0.5 represents an essentially worthless marker. A level of *p* < 0.05 is considered statistically significant.

## Results

### The CD44+ CRC: a distinct phenotype

**Fig. 1:**
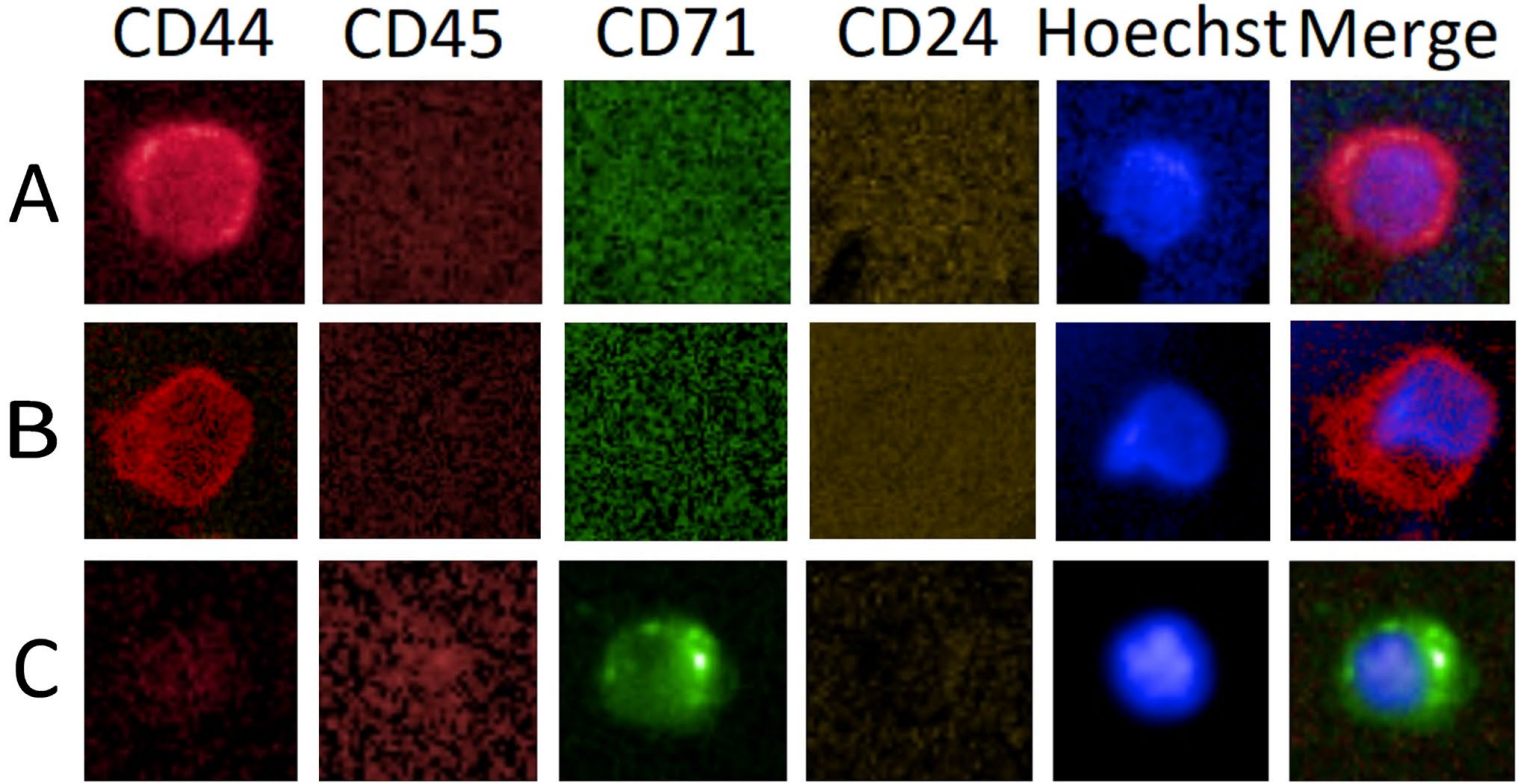
**A** Putative CD44pos/CD24neg phenotype negative for CD71, **B** an irregular and viable CD44+ cells allegedly associated with malignancy with a morphological index greater 35, and **C** a classic late erythroblast with CD71pos/CD44neg phenotype

The CD44+ CRC was found in all donor groups (Table 1) at various concentrations ranging from 0 to 46 cells per 5 mL and bares the question of cell identity. One might agree that an unambiguous positive identification of the cell type or types would be extremely cumbersome knowing that the antigen is widely expressed on hematopoietic and non-hematopoietic cells yet, alleging bone marrow origin. Therefore, we approached cell identification within capacity of our liquid biopsy platform based on the exclusion principle of cell types that are known to express the CD44 marker. The results may allow us to exclude circulating erythroblasts (CEB). CEB are represented by the CD71+/CD44±/24−/45− phenotype and are common in healthy and more so in afflicted individuals (Schreier, et al. 2021b). Findings of CD44+ CEB would allude to egress of earliest erythroblasts from the bone marrow as such allude to dysfunctional erythropoiesis (Schreier, et al. 2021b). The review of in total 54 CD44+ CRC that were co-stained according to the CD71+/CD44±/24−/45− phenotype and derived from CD44+rich samples Nr. 1.4 and Nr. 13 suggested that the CD44+/24− phenotype does not include erythroid cell events. None of the 54 CD44+ cells showed CD71 expression. Such findings are illustrated in Fig. 1. When scrutinizing CD44 status in erythroblasts, the individual afflicted with diabetes (sample Nr. 13) counted 34 erythroid CD71 positive events (data not shown) and revealed 4 CD44dim events with a maximum CD44 intensity-to-background ratio of 1.25 × and none showing a distinct membrane staining similar to CD71. A similar finding was obtained from the analysis of cancer patient (sample Nr. 1.4) having counted 8 erythroid events and none showing recognizable CD44 expression. In contrast, only 13 out of all 233 or 5.6% of the CD44+ CRC showed similar dim CD44 intensities when compared with the erythroblasts and additionally, supports the conclusion of a distinct cell phenotype.

**Table 1:** Sample data

Sample class	Sample Nr.	Sample description/age	CD44+ CRC	EpCam+ CRC
Negative control—healthy	14	Male/55	3	None
	16	Male/41	1	None
	17	Male/39	0	None
	18	Female/36	0	None
	20	Female/39	1	None
	21	Female/50	2	None
	22	Male/33	3	None
	23	Female/36	2	None
	25	Female/39	2	None
	28	Male/41	1	None
		Median	1.5 ± 1.08	
Negative control—non-malignant disorder	13	Diabetes type II, insulin treatment, female/55	46	None
	15	Pfizer 1st inoculation 1 week after, female/36	11	None
	19	Mild non-alcoholic fatty liver, male/48	9	None
	29	Self-limiting endothelial dysfunction/42	0	None
		Median	10 ± 20.24	
Positive control—treatment-naive	1.1	Female 66, T2 lobular carcinoma, pleomorphic and solid variant, moderate diff. Hormone-positive phenotype pre-surgery sample	15	17
	1.2	Post-surgery sample	19	2
	5.1	Female 37, T2 moderately diff. invasive ductal carcinoma, triple negative phenotype/post-surgery sample	4	21
	7	Female 69, T1 high diff. invasive ductal carcinoma, hormone positive, post-surgery sample	8	1
	9.1	Female 49, T2 moderately diff. invasive ductal carcinoma, hormone positive, Her2+ phenotype, post-surgery sample	15	0
	12	Female 39, T2 moderate diff. invasive ductal carcinoma, hormone-positive phenotype, post-surgery sample	2	0
	8	Female 66, T1 moderately poor diff. invasive ductal carcinoma, hormone-positive phenotype, follow-up sample 6 months, no adjuvant treatment	7	0
	26	Female, 62, T2 invasive ductal carcinoma, LN involvement 1 in 12, hormone positive, Her2 10% phenotype	9	0
	27	Female 63, local recurrence patient 15 years, T2	0	7
		Median	8 ± 6.44	
Positive control cohort—treatment mid-term	1.3	Mid-term	9	0
	5.2	Mid-term	11	11
	9.2	Mid-term	19	0
		Median	11 ± 5.29	
Positive control—completion	1.4	Completion	8	0
	5.3	Completion	10	1
	9.3	Completion	17	4
Sum		Median	10 ± 4.73

**Fig. 2:**
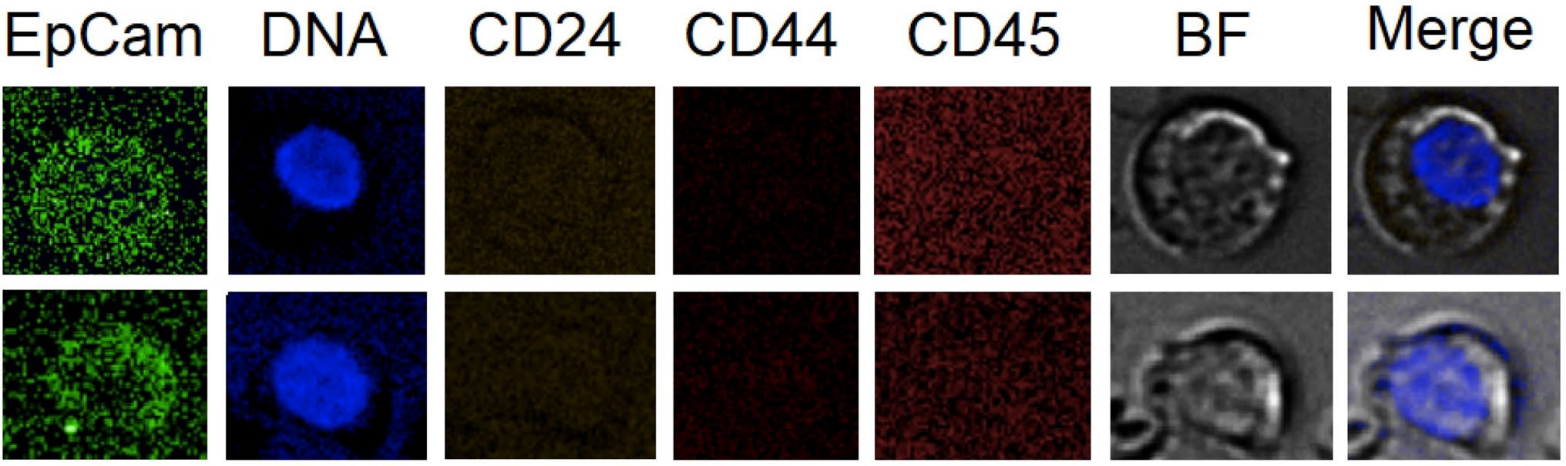
Two examples of circulating epithelial cells detected in post-surgery sample and obtained from donor sample Nr. 5.1

Furthermore, we investigated the CD44 status in EpCam+ /CD45− CRC in the cancer patient cohorts following the idea of detecting cancer stem cells. A total of 64 events were recorded (average 6.1 cells per 5 mL, *n* = 15), yet none showing positive expression of CD44 (Fig. 2). Therefore, cell findings of EpCam+ and CD44+ CRC did not overlap which may not exclude the likelihood of the presence of cancer stem phenotype at a very small fraction, yet suggesting that the CD44+ CRC identified in the donors are different to CTC and CEB.

### CD44+ CRC: cytological abnormality above 1 cell per mL

The results may support clinical usability of the CD44+ CRC cell with EpCam−/CD71−/CD44+/24−/45−/DNA+ phenotype as a biomarker to detect systemic inflammation. Our data suggest that the CD44+ CRC has its place among the common rare cell population as a separate cell entity which is supported by the finding of the respective phenotype in 8 out of 10 healthy individuals measuring a median 1.5 cells per 5 mL. The identified concentration level denotes a background concentration at healthy physiology. Therefore, CD44+ CRC could be used to predict healthiness when detected at baseline concentration levels in recovered or otherwise formerly afflicted individuals. Statistical significant marker elevation when referenced to the healthy cohort is supported by our results (*p* < 0.008) measuring a median concentration per 5 mL of 10 cells with SD ± 20 cells (*n* = 3) (0–46 cell range across samples) in the non-malignancy afflicted cohort and a median of 9.5 cells per mL SD ± 5.3 across all samples (*n* = 15) (0–19 cell range across samples) in the cohort of early-stage breast cancer sample. Therefore, elevation of the CD44+ CRC suffices to evidence cytological abnormality above a certain cutoff concentration that is apparently associated with a disorder in the most general interpretation. A suitable cutoff value to allege abnormality was deduced based on the limit of detection (LOD) determination following the equation LOD = mean (limit of blank) + 3xSD. The limit of blank is represented by the healthy cohort data (*n* = 10) measuring a mean concentration of 1.5 ± 1.08 CD44+ CRC per 5 mL. Accordingly, the LOD measured 4.5 cells per 5 mL, then choosing the cutoff to declare abnormality to measure 5 cells per 5 mL for our liquid biopsy platform. Therefore, cell findings below 5 CD44+ CRC per 5 mL would indicate physiological normality even though, one could make a claim of a pathological interpretation of very low inflammatory situation.

### CD44+ CRC subtyping based on morphological index

The hypothesis of the association of the CD44+ CRC specificity with residual cancer disease must be rejected in view of our finding of similar median elevation in the afflicted groups (non-malignant and malignant) when compared to the healthy cohort thus, evidencing no specificity to any of the health conditions tested and ultimately, excluding the prospect of predicting malignancy by the CD44+ CRC based on quantification of the phenotype. Therefore, a strategy to increase specificity was needed and commonly achieved based on increased identification depth. Greater identification depth could be realized by cyto-morphological subtyping. Morphological variability was apparent, leading us to investigate the correlation of cytological features within the CD44+ CRC with malignancy. A view on the appearances of the CD44+ CRC by a cytologist would reveal at instant three major features, that included membrane staining intensity of the CD44 marker, the nucleus fluorescence intensity and the shape of the cells. Therefore, we intended to transform those three morphological features into a quantifiable measure referred to as morphological index using the maximum intensity of CD44 and Hoechst staining and the shape for each cellular event with the latter being quantified by binning shapes into three categories numbered 0, 1 and 2 being round, oval or less round and irregular, respectively. The morphological index was calculated by multiplying the values of the two intensity parameters of CD44 and Hoechst being fluorescence intensity ratios (see methods) with the squared shape nominator. The shape nominator was squared given highest prominence of cell change when compared to pixel intensities and resulting in a morphological index for each cell ranging from 1.4 to 2794. The lowest value was found commonly in healthy (Fig. 1A) and the highest only in diseased individuals (Fig. 1B). Area under the ROC curve analysis served the purpose of evaluating the association of the morphological index with malignancy. We compared morphological indexes of CD44+ CRC from 8 chemotherapy-naive cancer samples representing the true-positive samples and counting 79 cells with all 12 non-malignant afflicted samples including healthy cohort representing the true negative cohort and counting 80 cells. The AUC analysis supported an association of increased morphological index with malignancy and identified the morphological index as optimal cutoff having 59% sensitivity and 86% specificity in the early-stage cancer setting [AUC = 0.784 (95% CI: 0.7025 to 0.8445]. The cutoff value identification followed the highest Youden Index resulting in the morphological index ≥ 17.33. Using this cutoff, a PPV of 0.81 and a NPV of 0.68 was determined. A morphological index lower 17 represents cells either being round or low in fluorescence intensities. Therefore, the analysis suggests that the morphology of a certain fraction of CD44+ CRC in malignancy samples is significant different with respect to the three cytological features that comprise the morphological index. This fraction of CD44+ CRC was of further interest as presumes a stronger association with malignancy and could be further specified by increasing the morphological index cutoff value to 35. Accordingly, the sensitivity dropped to 43% in favor of a specificity increase to 94%. In consequence, a morphological cell class could be identified that was irregular in shape, having intact and active nucleus with varying CD44 staining intensities. The cell subtype was denoted as CD44_i35_ in this context and would be declared as useful marker to predict residual cancer disease associated with systemic inflammation with a PPV of 0.87 and a NPV of 0.62.

### Proof of concept of the CD44_i35_ marker

According to the preceding results, the inflammation marker CD44_i35_ and the quantitative cutoff criterion (> 5 cells per 5 mL) denote a criterion of rare cell abnormality and in combination, can be used to evidence disorder in an individual and potentially predict persisting residual disease-associated systemic inflammation in early-stage cancer with a specificity of 94% applied either after surgery or ACT. We verified the criterion of rare cell abnormality in retrospective on our data set (Table 2). As could be expected, all healthy individuals were negative. CD44+ CRC quantity above cutoff was detected in three out of four samples of the non-malignant group. The diabetes patient (sample 13) and the freshly vaccinated person (sample 15) were cytological abnormal measuring 2 CD44_i35_ out of 46 CD44+ CRC and 1 CD44_i35_ out 11 CD44+ CRC, respectively. Therefore, a false-positive rate of 5.2% was calculated (CD44_i35_/CD44 ratio) and could be expected according to the 94% specificity of the marker. In the cancer patient samples, we would expect a similar high percentage of CD44+ CRC elevation when compared to the noncancer afflicted cohort yet, a marker increase in CD44_i35_ marker. Accordingly, the percentage of rare cell abnormality by quantity measured 80% and the frequency of CD44_i35_ fraction within CD44+ CRC (true-positive fraction) in each cancer cohort was significantly increased measuring on average 41.7% (Table 2). A clinical sensitivity of the CD44_i35_ marker greater 78% may be deduced given the finding of seven positive out of nine treatment-naive post-surgery patients.

**Table 2:** Validation of rare cell abnormality criterion

	Healthy	Non-malignant	Pre-treatment cancer	Mid-treatment cancer	Treatment completion cancer
Abnormality by cell quantity	0/9	3/4	6/9	3/3	3/3
Abnormality by Cd44_i35_	0/9	2/4	7/9	3/3	3/3
CD44_i35_/CD44+ CRC ratio	Not relevant	5.2%	41.8%	46.1%	37.1%
Average morphological index	15.7	12.8	40.0	114	221

### Marker behavior as objective response measure

The use of the CD44+ CRC and CD44_i35_ as objective response marker for ACT might be of interest. The findings of sustained CD44+ CRC and CD44_i35_ in a similar quality upon therapy and completion suggest a continued pathological status in post-surgery breast cancer patients. Despite a small sample volume, a clear reduction in cell numbers could not be observed in the three follow-up patient samples with patient 1 showing a decline, patient 5 an increase and patient 9 a stable situation suggesting that a more complex situation influences response dynamics of CD44+ CRC across therapy and thereafter. The data would suggest that the average morphological index of CD44_i35_ increases during therapy and thereafter (Table 2) measuring a clear difference in value of 164.7 and the 39.7 on average for the treatment and treatment-naive samples, respectively. We, therefore, asked the question, if the systemic treatment is likewise evoking CD44_i35_ elevation by comparison of CD44+ CRC data sets from the 8 non-treated cancer samples counting 79 cells declared as true negative with in total 6 samples including the treated mid-term and the completion cohort counting 78 cells, declared as true positive using AUC analysis. The analysis confirms a slight association of increasing morphological index with chemotherapy and the completion thereof and identified the morphological index as optimal cutoff having 22% sensitivity and 97% specificity, [AUC = 0.564 (95% CI 0.462–0.651, *p* = 0.0483, *z*-value = 1.32]. The cutoff value identification followed the highest Youden Index resulting in the significantly increased cutoff value for the morphological index ≥ 146.8. Using this cutoff, a PPV of 88.9% and a NPV of 54.8% for effects by ACT were determined. Therefore, the results support the conclusion that ACT affects the CD44+ CRC quantity and quality, allow a good prediction of ACT side effects yet, and do not support the use of CD44_i35_ as objective response marker.

## Discussion

The lack of wholesome and sufficiently sensitive objective assessment of residual cancer disease in early-stage breast cancer patients causes several problems that include the false identification of high-risk patients as complete responders, the inability to predict benefit of adjuvant chemotherapy, the inability to evidence and predict harmful side effects by systemic chemotherapy and particularly the inhibition of development and validation of specialized and/or new adjuvant treatment modalities. Cell-based and ct-DNA liquid biopsy platforms are under investigation to fill the gap and may appear most suitable for MRD assessment in general. However, we argue that the current marker practice is unfit for purpose in early-stage cancer patients. As already introduced, there is little benefit in using circulating tumor-derived markers expecting and in fact, reading a low MRD positivity rate across respective independent liquid biopsy platforms (Pantel and Alix-Panabières 2019; Parsons et al. 2020). Apparently, the low clinical sensitivity for MRD detection is accepted in the field, yet does not provide a high resolution of MRD and obviously, does not stringently correlate CTC negative with MRD-negative status (Aceto 2019). Nevertheless, the marker behavior translated in parts into acceptable positive predicted values of relapse (Pantel and Alix-Panabières 2019). Of note is that marker validation used follow-up periods that mostly did not exceed 5 years. Problematic is the late relapse in MRD-negative early-stage cancer patients 10 years onwards, suggesting a limited benefit for a majority of early-stage breast cancer patients when using current liquid biopsy platforms and argue that improvement is achieved in general by more sensitive markers towards residual cancer. We also would like to bring to the attention a perplexing situation in CTC-based liquid biopsy platforms failing to agree on the positive correlation of the circulating epithelial cell marker with tissue tumor burden and consequently, questioning the specificity towards malignancy and independence from therapy effects. In fact, evidence has been provided for the influence and thus, dependency of CTC concentration on ACT modes (Shliakhtunou 2021). An over-claim of malignancy in MRD could impede evaluation of therapy efficacy, put follow-up patients under high anxiety and eventually unnecessary treatments and provide a wrong feedback for pharmacodynamic investigations. Explanations of MRD persistence at high level and even elevation of CTC in the post-surgery setting given by those operating more sensitive platforms relate to CTC showers after surgery (Pachmann 2015). The explanation would imply a sudden emergence of a long-lived, viable and activated, highly proliferative yet, non-aggressive CTC type after surgery in early-stage cancer patients with a certain risk of relapse only several years after treatment completion triggered by surgery. As it appears, the controversy represents a contradiction between the theory of tissue egress, CTC half-life and CTC shower persistence and might be ascribed to a mere lack in marker standardization and improper cohort validation (Geeurickx and Hendrix 2020). It is compelling to us that groups which omit a stringent and high sensitive measure of the hematopoietic status of circulating epithelial cells (Shliakhtunou 2021; Schott et al. 2021) and those simply including CD45 positive status as CTC (Papadaki et al. 2020; Park et al. 2020; Zahid et al. 2018) seem to achieve higher MRD positivity rates and CTC levels when compared to platforms that follow the original definition of epithelial CD45−/Dapi+ cells (Pantel and Alix-Panabières 2019; Allard et al. 2004). The controversy may also be underpinned by the choices of control cohorts. We observe, that overly high sensitive platforms seem to lack validation scrutiny then, failing to include non-malignancy afflicted cohorts. In contrast, CellSearch used a non-malignant cohort that included fibrocystic disease, fibroadenoma, ductal hyperplasia, and microcalcifications diabetes, arthritis, asthma, thyroid abnormalities, and hyper-cholesterolemia and concluded with 99.7% specificity (Allard et al. 2004). Still, one could argue that this high specificity might change depending on the non-malignant cohort type for example including more chronic inflammatory diseases (Rosenbaum et al. 2017; Pantel et al. 2012).

In view of shortcomings of current liquid biopsy markers to unambiguously detect MRD, the in-depth investigation of occult MRD with respect to tumor-associated systemic inflammation as an alternative and perhaps more pronounced aspect of breast cancer MRD was of interest in this work. The prospect would be the contribution to grade residual cancer in every patient rather than to stratify MRD into positive or negative and thus, could hold marked potential to improve relapse prediction and provide meaningful follow-up scheduling. Ideally, circulating cellular inflammation markers would be needed in a systemic disease. Therefore, this initial prove of concept study investigated the association of the CD44+ CRC with malignancy. The marker purpose would relate to the detection of residual CD44+-associated systemic inflammation in early-stage breast cancer patients after surgery and after completion of adjuvant therapy. It should be noted that the effort of associating the phenotype with malignancy in breast cancer MRD was independent of the distinct cytopathological identification of the cell type. Knowledge of the cell identity may support diagnostic interpretation of the finding. However, an unambiguous identification of the CD44+ CRC was unachieved using the less isoform-specific CD44 clone IM7 as to increase sensitivity towards the antigen and the given liquid biopsy platform set up and denotes a separate research effort. Nevertheless, our results support the notion of a rare cell type that is distinct from circulating tumor stem cells or circulating erythroblasts and is apparently common to the healthy rare cell population. The CD44+ CRC appeared perfectly round, with low CD44 expression, was low concentrated yet, remarkable frequent already in healthy donors. We theorized having detected an inactive stromal progenitor cell type with a purpose of patrolling the body via the blood stream and undergo apoptosis while circulating, if not needed. In the contrary case, the cells become activated effector cells as can be evidenced by the morphological alteration and increase in CD44 expression as well as higher DNA staining. Expression changes of the kind are not unknown to research with mesenchymal cells (Herrera et al. 2007; Qian et al. 2012).

The CD44+ CRC was found in all cohorts and thus, lowers the prospect of sufficient specificity towards malignancy based solely on phenotyping. As already introduced, the multi-pathology association of CRC in general, poses a high risk of overclaiming a pathological status related to malignancy by the CD44+ CRC marker. A workable solution to increase specificity is subtyping. The approach of increasing phenotype identification-depth or adding molecular analysis, such as FISH is most common, yet complicates methodology and/or is heavily on costs of sensitivity. Cytological feature analysis may provide a suitable alternative and is tapping into a rich pool of diagnostic information, since morphological changes are directly underpinned by cellular activities, molecular abnormalities and functions. However, subtyping by morphology is uncommon in liquid biopsy most probably due to issues in standardization and transferability. The phenotype seemed to include a wide range of cellular appearances that was found useful in probing the association with cancer. In that regard, we introduced a quantifiable measure of cell morphology and intensity referred to as morphological index comprising a minimal set of cytological features that included the cell shape, CD44 and Hoechst intensity-to-background ratio. The three cytological parameters were chosen according to most obvious differences in cell appearances. Interestingly, the morphological index varied from a single digit to triple digit numbers thus, showing a high resolution of the cellular appearance and enabled a detailed AUC analysis. The statistical analysis supported our initial impression of a positive correlation of morphological index with disorder severity and allowed the identification of a morphological index cutoff for malignancy-associated inflammation that measured 35 hence, the name CD44_i35_. We shall note that the CD44_i35_ marker does not enable the diagnosis of malignancy. We gained the understanding that CD44 inflammation is independent of a specific malignancy type or status, yet malignancy is highly associated with CD44 inflammation. This would explain the finding of a significant influence of systemic therapy on the morphological index that was increased when compared to treatment-naive patient results, suggesting that systemic adjuvant standard care elicits respective inflammatory host response and affects the marker behavior. In consequence, CD44_i35_ is unfit for objective response measurement in ACT warranting deeper investigations about effects of systemic chemotherapy and their recovery. On the bright side, the outlook of predicting treatment-associated systemic inflammation can be given as a monitoring practice using the CD44_i147_ marker. The actual biomarker application that seemed to be supported by this validation study is centered around the better understanding of the disease status. Therefore, the outlook as surrogate marker for objective measure of therapy success and recovery can be given and expecting receding CD44+ CRC levels below the healthy cutoff level only in fully recovered patients. We have seen in three follow-up patients 1, 5, and 10 that this may not be always the case. In addition, patient 7 was followed up 6 months after surgery having received only hormone therapy showing sustained CD44+ inflammation. Of limitation might be that systemic inflammation related to malignancy cannot be included nor excluded in case of positive findings of CD44+ CRC abnormality but negative CD44_i35_ detection in post-surgery patients. Often a comorbidity accompanies the cancer patient, which could be the source of the finding.

The finding of a physiological base line concentration below the cutoff of 5 cells per 5 mL whole blood suggests greater sensitivity of the liquid biopsy platform in use and supports the before mentioned idea of grading inflammatory MRD from a healthy status till severe illness. It shall be noted herein that this cutoff naturally depends on the functional sensitivity of the liquid biopsy platform. In this regard, not every early-stage patient was CD44_i35_ marker positive. The cohort volume was too small and only allows a careful prediction of the clinical sensitivity of CD44−related MRD in post-surgery patients, yet suggesting improved sensitivity of at least 78% when compared to non-hematopoietic tumor-derived markers. Nevertheless, the comparatively high sensitivity may support the development as predictive marker of relapse and a marker to monitor anti-inflammatory therapies in the adjuvant therapy setting.

## Conclusion

We intended to develop a workable inflammation marker in cell-based liquid biopsy to predict malignancy based on a CD44-related circulating rare cell phenotype as an alternative to probe a perhaps more exposed aspect of cancer residual disease. CD44 is a major participant to drive inflammation by supporting cellular interaction and cell activation. The CD44+ non-hematopoietic phenotype was found conserved among the rare cell population in healthy donors and elevated non-specifically upon various pathologies with systemic involvement, concluding with multi-pathology association underpinned by systemic inflammation. Standard AUC analysis was used to identify a CD44+ CRC morphological subtype with greater specificity towards malignancy. We concluded with improved clinical usefulness of the novel marker with respect to grading inflammation in cancer patients and predicting residual cancer disease in post-surgery and/or post-ACT patients. The marker failed to be useful for objective response measurement during ACT having encountered influence of standard ACT on systemic inflammation. In combination with other rare cell markers, a wholesome picture of systemic MRD can be provided and exceeds diagnostic information when compared to a biopsy. A relevant panel would include CTC to measure residual tumor burden, circulating erythroid cells to measure bone marrow damage, circulating endothelial cells to measure vascular damage and recovery, and CD44+ cells to measure a general cellular state of activation.

## Data Availability

Not applicable.
